# Variation of Metallothionein I and II Gene Expression in the Bank Vole (*Clethrionomys glareolus*) Under Environmental Zinc and Cadmium Exposure

**DOI:** 10.1007/s00244-017-0485-7

**Published:** 2017-12-16

**Authors:** Magdalena Mikowska, Barbara Dziublińska, Renata Świergosz-Kowalewska

**Affiliations:** Institute of Environmental Sciences, Jagiellonian University, Gronostajowa 7, 30-387 Kraków, Poland

## Abstract

The main idea of the study was to assess how environmental metal pollution activates defence responses at transcription levels in the tissues of bank voles (*Clethrionomys glareolus*). For this purpose, the metallothioneine (MT) genes expression (a well known biomarker of exposure and response to various metals) was measured. The real-time PCR method was used for relative quantification of metallothionein I and metallothionein II expressions in the livers, kidneys and testes of bank voles from six populations exposed to different contaminants, mainly zinc, cadmium and iron. The assessment of Zn, Cu and Fe concentrations in the tissues allowed to study the MTs gene expression responses to these metals. ANOVA analysis showed differences between populations in terms of metal concentration in tissues, livers and kidneys. Student *T* test showed significant differences in metal concentration between unpolluted and polluted sites only for the liver tissue: significantly lower Zn levels and significantly higher Fe levels in the unpolluted sites. Kruskal–Wallis test performed on *C*_T_ data shows differences in the gene expressions between populations for both MT genes for liver and testes. In the liver metallothionein I gene expression was upregulated in populations considered as more polluted (up to 7.5 higher expression in Miasteczko Śląskie comparing to Mikołajki). Expression of metallothionein II revealed a similar pattern. In kidneys, differences in expression of both MT genes were not that evident. In testes, MT upregulation in polluted sites was noted for metallothionein II. For metallothionein however, we found downregulation in populations from more contaminated sites. The expressions of both MTs were positively influenced by cadmium in kidney (concentration data from the previous study) and zinc and copper in liver, while cadmium had effects only on the liver MT II gene expression. Positive relationship was obtained for lead and metallothionein II expression in the liver.

Metal pollution continues to pose a serious problem, especially for organisms that inhabit areas near sources of metal contamination. Although many actions have been undertaken to decrease metal emission and environmental contamination, wildlife is still at risk (Bickham et al. 2000). To better describe animal functioning in contaminated habitats, metal tissue assessment approaches themselves are not enough. Data concerning other biomarkers that show responses at different biological organisation levels are useful—starting from the cellular level (e.g., markers such as glutathione concentration) (Srikanth et al. 2013) to population level (e.g., biomarkers of genetic diversity) (Guban et al. 2015). Due to this, contemporary biology has developed several tools to study different types of biomarkers (Shugart 2000; Walker et al. 2001; Handy et al. 2003).

When facing a contaminated environment, organisms activate a number of defence mechanisms, such as reduction of absorption, effective excretion, immobilisation in cell granules, or production of binding molecules. The first molecules that respond in the defence are metallothioneins (MTs), proteins taking part in metal scavenging (Sigel et al. 2009). Four groups of MTs have been described for mammals, among which MT I and MT II are commonly found in various tissues (Andrews 2000), whereas MT III and MT IV are more tissue-specific. Metallothioneins have been widely described among terrestrial vertebrates (Day et al. 1981), invertebrates (Roesijadi 1996), and plants (Cobbett and Goldsbrough 2002). MTs in higher eukaryotes regulate zinc and copper levels and its distribution within the cell and organism (Bremner 1979; Nordberg 1998). These low-molecular weight proteins are rich in cysteines, which increase their capacity for scavenging of metal ions, such as Zn, Cd, Cu, Ag, and Hg (Miles et al. 2000; Wang and Fowler 2008). Their protective role against cadmium toxicity and other oxidative stress is well known (Andrews 2000). Previous studies have shown that metal stress is a good MT inducer (Świergosz-Kowalewska et al. 2007). The strength of this defence depends on several factors connected with the metal characteristics and organism attributes. The literature also shows that metallothioneins may be induced by nonmetallic toxic agents (glucocorticoids) and physiological conditions, such as nutritional status or pregnancy (Gil and Pla 2001).

Metallothionein has been used as a biomarker of metal exposure in several investigations (Amiard et al. 2006; Atli and Canli 2008; Espinoza et al. 2012); however, the Cd-saturation method (Onosaka and Cherian 1982) was implemented more frequently; it is less costly and laborious than the gene expression method (real-time polymerase chain reaction [RT-PCR]) used in our studies. Research shows that Cd–MT, Cu–MT, and Zn–MT fractions in the kidneys and liver increase with increasing cytosolic metal concentrations (Rogival et al. 2007). Our approach focused on this specific detoxification process, because it may help to decrease metal toxicity through binding them in stable molecules and then facilitating effective excretion. It is not well documented how metallothionein gene expression varies in natural populations of rodents. Additionally, valuable data that show variation of this parameter in wild rodent populations would serve in future studies on bank vole populations.

As a result, we assessed metallothioneins gene expressions (MT I and MT II) in the tissues of wild bank voles (*Clethrionomys glareolus*), originating from metal polluted and unpolluted areas. The chosen study sites have a long history of smelting and mining and are still at risk from metal pollution (Łaszczyca et al. 2004; Augustyniak and Migula 2000). The tissue-dependent intensity of MT I and MT II gene expressions and the effects of environmental realistic exposure on this process are estimated. We hypothesised that the level of MT I and MT II gene expression would be correlated positively with increasing metal concentrations in the tissues, both essential (Cu, Zn, Fe) and toxic (Pb, Cd—data from our previous study, Mikowska et al. 2014), as measured in the kidneys and livers.

We chose the RT-PCR method, because we wanted to check the MTs defence responses at a transcriptional level, more connected with current exposure to pollution, and show the potential to activate this detoxifying mechanism.

## Materials and Methods

### Study Sites and Animal Trapping

The animals collected for this study originated from six study sites located in the northern and southern part of Poland. Three populations (Mikołajki, Teleśnica Oszwarowa, and Niepołomice) inhabited unpolluted sites; the other three (Miasteczko Śląskie, Katowice and Olkusz) were located in the vicinity of zinc/lead smelters in Southern Poland in the heavily metal-polluted Silesia region. Based on the information concerning the industrial operations, the sites were classified as unpolluted and polluted. Detailed information on the location of the sites was presented by Mikowska et al. (2014).

The animals were trapped during early autumn using a standard live-trapping method (Southwood and Henderson 2000) and were transported to the laboratory, where they were killed by decapitation and dissected according to standard procedure. The livers, kidneys, and testes were collected and homogenised. Part of each tissue was frozen at – 70 °C and kept for chemical (metal tissue contents) analyses. The other part of each tissue, approximately 30 mg, was stored in RNA*later* buffer (Sigma Aldrich, St. Louis, MO) in − 70 °C for gene expression analysis.

### Metal Analyses in the Tissues

Tissue samples (kidney and liver) were dried at 70 °C until they reached constant weight. Next, they were wet-digested in nitric acid (Suprapur; Merck, Darmstadt, Germany). The metal tissue contents (Cu in the liver; Zn and Fe in the liver and kidneys) were determined with atomic absorption spectrometry (AAnalyst 800; Perkin-Elmer, Waltham, MA). The copper concentration in the kidneys was not assessed due to technical limitation (a low amount of sample material). Certified reference material was used to check the analytical precision that was achieved (SRM 1577c Bovine Liver; National Institute of Standards and Technology, Gaithersburg, MD). The metal concentration is presented in units of metal milligrams per kilogram of dry weight tissue (mg kg^−1^ dw). Concentrations of Cd and Pb in the liver and kidney samples were measured in our previous study (Mikowska et al. 2014).

### RNA Isolation and Reverse Transcription

RNA was isolated using RNeasy Mini Kit (Qiagen, Hilden, Germany) from the livers, kidneys, and testes (according to the vendor’s protocol) from tissues stored in RNA*later* buffer at − 70 °C.

Approximately 30 mg of each tissue was manually homogenised. Isolation steps were performed using microcentrifuge (Centrifuge 5424, Eppendorf, Hamburg, Germany) with the maximum speed of 14,650 rpm. RNA was eluted with 50 µL of nuclease-free water (Qiagen) and stored at − 70 °C. RNA quality (absorbance ratio A260/A280), and quantity were verified using the NanoDrop ND-1000 spectrophotometer (PEQLAB Biotechnologie GmbH, Erlangen, Germany).

### Real-Time Polymerase Chain Reaction

RT-PCR was performed by using the QuantiFast SybrGreen RT PCR Kit (Qiagen) according to the protocol. The reactions were conducted with a 7500 Fast RT-PCR instrument (Applied Biosystems, Foster City, CA). The housekeeping gene 18S was used as a reference gene. Primer’s sequences for metallothionein I and II and 18S known from previous studies by Świergosz-Kowalewska et al. (2007) were synthesised by Genomed (Warsaw, Poland) and then used for gene expression analyses. To amplify the target genes, 20 µg of RNA from each tissue sample was used. At the end of each RT-PCR reaction, the melting curve procedure was performed to check for possible unspecific products. All the reactions were run in three replicates. On each plate one identical sample (SS—RNA isolated from one of random chosen bank vole liver samples) was run to standardise readings between plates. The results of MT I, MT II, and 18S gene expressions were determined as *C*_T_ (cycle number) values.

### Statistical Analysis

The means and standard error (± SE) were determined for the metal contents in the kidneys (zinc and iron) and livers (zinc, iron, and copper). The data were log transformed to fit normal distribution. The tissue metal levels between populations were compared by using one-way ANOVA (using the Statistica ver. 10 software package; StatSoft Inc., Tulsa, OK). The *T* test was conducted to compare metal levels in the tissues of the bank voles from the two types of populations: unpolluted site populations (Mikołajki, T. Oszwarowa, Niepołomice) and polluted site populations (M. Śląskie, Katowice, Olkusz).

The obtained the *C*_T_ value for the SS sample, which was located on each analysed plate, was used to standardise all of the *C*_T_ readings (Microsoft Office Excel 2003; Microsoft Corporation). Then, Δ*C*_T_ for each sample was calculated as a difference between the *C*_T_ value of the target gene (MT I or MT II) and the *C*_T_ value of the endogenous control (18S). The values of ΔΔ*C*_T_ were determined based on Δ*C*_T_ of one sample from the Mikołajki population. This population was set as a reference base for low concentrations of lead and cadmium in the tissues obtained in the previous study (Mikowska et al. 2014), as well as the expected low exposure to other pollutants, which might be conditioned by its location in a region of low urbanisation level. Relative expression was calculated as RQ = 2^−ΔΔ*C*т^. The mean RQ for the Mikołajki population was set as a reference expression = 1 to calculate the multiplication ratio for the other populations (Microsoft Excel 2003).

Comparisons between the populations in terms of gene expressions (Δ*C*_T_) were done with the Kruskal–Wallis test (Statistica ver. 10). Regression analysis was performed for each tissue between metal concentrations and Δ*C*_T_ values of MT I or MT II.

## Results

### Metal Content

Mean Zn levels in the liver tissue ranged from 88.8 mg/kg dw for animals from the T. Oszwarowa site to 128.0 mg/kg dw for the animals from the Katowice site (Table 1). For this tissue, the average Fe concentration was the highest in the bank voles from Mikołajki (924.5 mg/kg dw) and the lowest in the Katowice polluted site (494.3 mg/kg dw). Mean copper concentration in the livers of animals from all the studied populations was comparatively low: range 15.3–18.9 mg/kg dw.

**Table 1:**
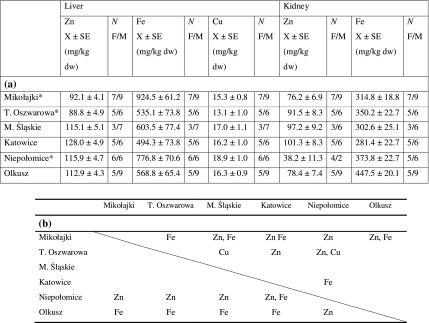
**a** Metal concentrations (in mg/kg dw) in the tissues of bank voles trapped in unpolluted (marked with *) and polluted sites. **b** Statistically significant differences in metal tissue concentration between sites (for liver: above the diagonal; for kidney: under the diagonal)

*X* arithmetic mean concentration, *SE* standard error, *N* number of individuals, *F* number of females, *M* number of males

*p* < 0.05

The highest average value for zinc concentration in the kidneys was noted for animals originating from Katowice (101.3 mg/kg dw). Analysis of metal concentration showed the lowest Zn levels in the kidneys of animals from Niepołomice (38.2 mg/kg dw). The Fe concentrations in the kidneys of animals ranged from 281.4 (Katowice) to 447.5 mg/kg dw (Olkusz).

The ANOVA analysis showed a variation between populations in metal concentration for both tissues—the liver and kidney (Table 1b). Results of the *T* test used to calculate the difference in metal concentration between the unpolluted and polluted sites was statistically significant only in the case of the liver tissue: significantly lower Zn levels and higher Fe levels in bank voles from the unpolluted sites (Table 2). The results also were statistically significant for lead and cadmium in the kidneys and liver, showing a high accumulation of both metals in animals originating from the polluted sites (Mikowska et al. 2014).

**Table 2:** Results of *T* test analyses (*p* value and *F* ratio) for differences in studied metal concentrations between unpolluted and polluted types of populations

Element/tissue	*p* value	*F* ratio
Zn liver	< 0.001	3.09
Fe liver	< 0.001	1.03
Pb liver*	0.014	10.54
Cd liver*	< 0.001	8.60
Pb kidney*	< 0.001	1.25
Cd kidney*	< 0.001	3.09

*Metal concentration data obtained in previous study (Mikowska et al. 2014)

### Gene Expression

**Fig. 1:**
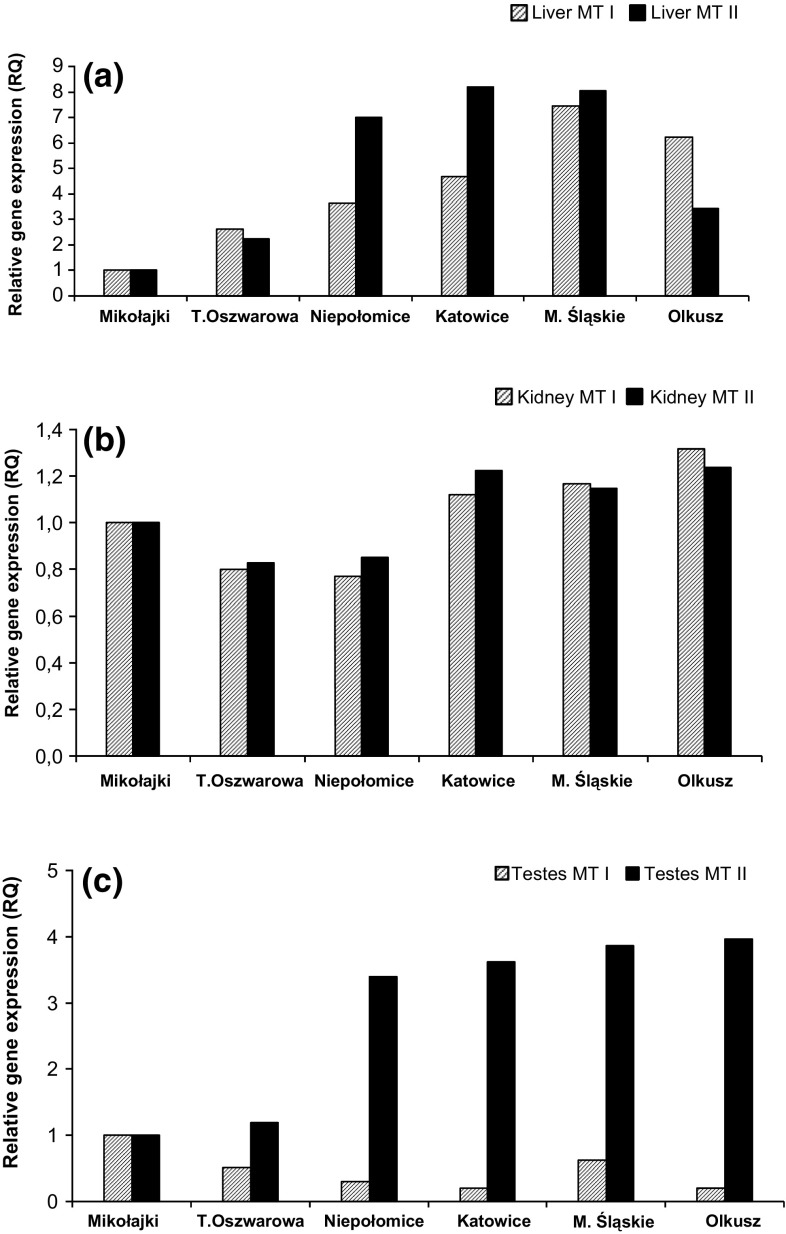
Normalised relative MT I/MT II gene expressions in the tissues of bank voles from unpolluted and polluted sites. Liver (**a**); kidney (**b**); testis (**c**)

The results of the Kruskal–Wallis test performed on Δ*C*_T_ data show statistical differences in genes expressions between populations in the case of both MT genes for the liver and testes (Table 3). The highest MT I expressions (RQ values) in the liver tissue of animals were found in the M. Śląskie polluted site, up to 7.5 time higher than in the Mikołajki site (Fig. 1a). Similarly, the MT II expressions were the highest in Katowice and M. Śląskie-up to eight times that of Mikołajki. For both MTs, the lowest values were obtained in the livers of animals from Mikołajki site.

**Table 3:**
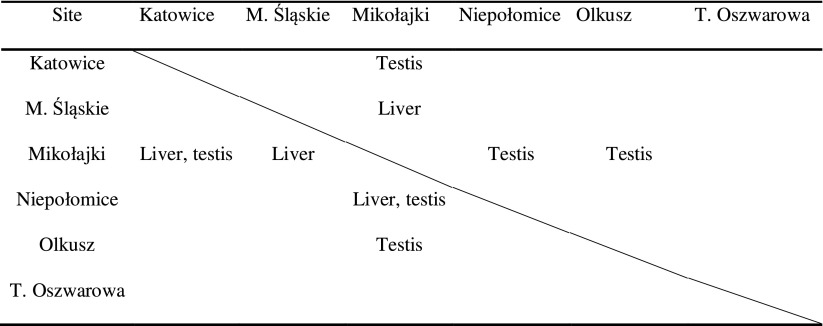
Significant between-population differences (*p* < 0.05) in expressions of MT I (above diagonal) and MT II (under diagonal) found for studied tissues

Expressions of both MTs in the kidney tissue were not such strongly regulated as in the liver (Fig. 1b). Both metallothioneins in the kidney tissues of animals from T. Oszwarowa and Niepołomice were downregulated compared to the Mikołajki site. The levels of MTs expressions in the other populations were around the levels obtained for the Mikołajki site.

The lowest level of MT I expression in the testes was found in the animals from the Olkusz and Katowice sites, whereas the highest was at the Mikołajki site (Fig. 1c). MT II expressions in the testes were more pronounced than in the case of MT I-up to four times higher in the Olkusz than in Mikołajki site.

**Fig. 2:**
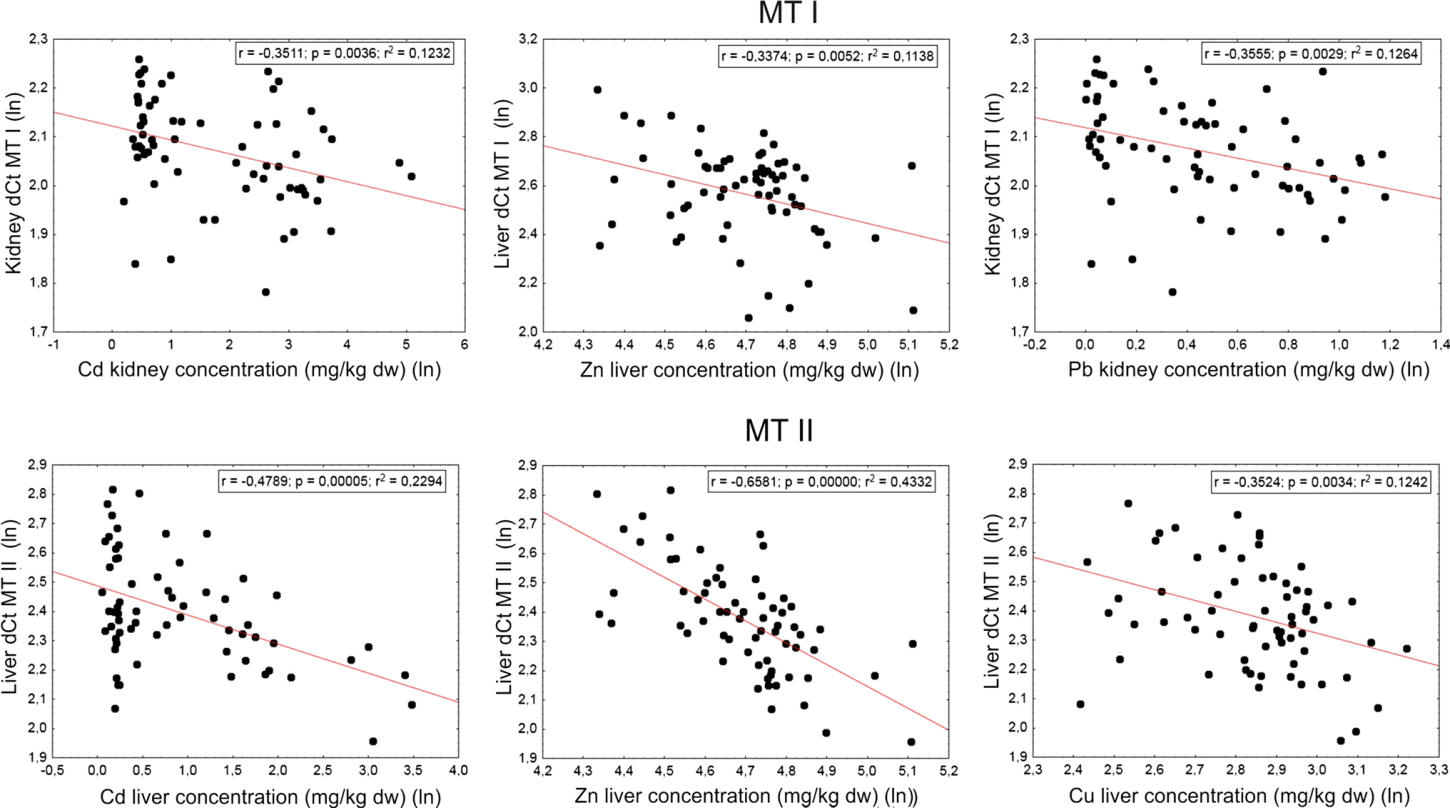
Relationships between metal concentrations (mg/kg dw) in the liver or kidney of bank voles and MT I or MT II gene expressions (expressed as Δ*C*_T_ values)

The results of the regression analysis showed a significant relationship between cadmium and lead concentrations and expressions of both MTs in the kidney (Table 4; Fig. 2). In the liver, we found major effects of zinc and copper concentrations on both MT I and MT II gene expressions, as well as between cadmium and MT II gene expression (Table 4; Fig. 2).

**Table 4:** Summary of regression analyses (*p* value, *F* ratio, and degrees of freedom) between the tissue metals concentrations and MT I or MT II gene expressions (based on Δ*C*_T_ values) relationship

		Pb	Cd	Zn	Cu
MT I	Liver	*p* = 0.015		*p* = 0.005	*p* = 0.025
		*F* = 6.215		*F* = 8.349	*F* = 5.284
		*df* = 67		*df* = 65	*df* = 66
		(−)		(−)	(−)
	Kidney	*p* = 0.003	*p* = 0.004		NA
		*F* = 9.548	*F* = 9.136		
		*df* = 66	*df* = 65		
		(−)	(−)		
MT II	Liver		*p* < 0.001	*p* < 0.001	*p* = 0.003
			*F* = 18.751	*F* = 48.905	*F* = 9.217
			*df* = 63	*df* = 64	*df* = 65
			(−)	(−)	(−)
	Kidney	*p* = 0.016	*p* = 0.018		NA
		*F* = 6.151	*F* = 5.878		
		*df* = 66	*df* = 65		
		(−)	(−)		

Only significant *p* values are reported

Directions of relationships are put in brackets (−)

*NA* concentration not measured, *df* degrees of freedom

## Discussion

The main goal of this work was to ascertain how animals cope with metal contamination in terms of transcriptional regulation of the MT I and MT II gene expressions. Both the results of metal concentrations and gene expressions (quantitative PCR results) studied in the same tissue samples allowed us to correlate exposure directly with the level of tissue MT defence and assess the potential effects of environmental contamination to these organisms. The uniqueness of this kind of study lies in an approach of assessing this biomarker in naturally exposed wild populations and also showing its natural variation. Because the literature shows that photoperiod may change the level of cadmium concentrations in organs (Włostowski et al. 2000) and also the level of metallothioneins—higher level of MTs in winter than in summer (Marques et al. 2008)—all animals were trapped during one season. In this way, data variance obtained in the study was not due to different photoperiods. By separately grouping the data for populations located near zinc–lead smelters (polluted sites) and those occupying relatively unpolluted sites, we could study not only the differences between single populations, but also the differences between two types of populations: unpolluted and polluted.

By choosing populations for the study from differently contaminated sites (for more detail, cf. Mikowska et al. 2014), we expected to find distinct differences for both metal levels and MTs expressions between them. Statistical analysis did not fully confirm our expectations and initially conducted site classification for unpolluted and polluted. Analyses of the differences between single populations showed that Zn concentrations in the livers of animals from Niepołomice, classified as unpolluted, differed from the other results for the unpolluted sites, and are in fact rather comparable to Zn levels obtained for the polluted sites (Table 1).

**Fig. 3:**
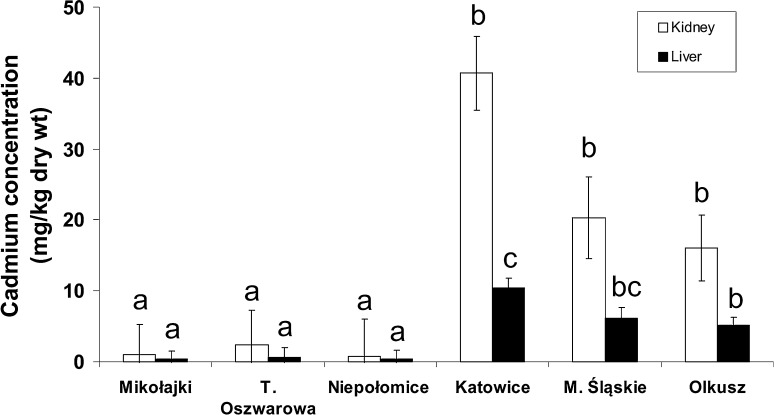
Cadmium concentrations (mg/kg dw) in the tissues of bank voles trapped in unpolluted and polluted sites (modified from Mikowska et al. 2014). Bars indicate standard errors. Different letters above bar indicate significant difference between populations separately for each tissue

Interesting findings concerning Fe levels in the livers, which are comparatively low at polluted sites, suggest iron depletion rather than enhancement. The lowest Cu concentration found in the livers of animals from T. Oszwarowa significantly differed compared with the values obtained for M. Śląskie and Niepołomice, but all of the values were within the range of natural levels (Mikowska et al. 2014). The zinc and iron levels identified in the tissues of the studied animals were comparable to those found in bank voles by Swiergosz-Kowalewska et al. (2007), and they are within a range of environmental variation typical for both unpolluted and polluted sites. The cadmium and lead concentrations in the liver and kidney of the studied animals were already published in a previous paper (Mikowska et al. 2014) and also reflected between-population differences in the levels of these metals (Fig. 3). These results confirmed our expectations about increased cadmium and lead levels in the tissues of animals inhabiting areas in close proximity to main sources of pollutants (Mikowska et al. 2014). Generally, the animal exposure to metals and their uptake in the polluted sites were higher than in the unpolluted sites.

As a response to lead and cadmium accumulation in the tissues of bank voles, the study clearly showed between-population differences in MT I and MT II expression for the livers and testes of the animals but not for the kidney tissue (ANOVA test). Between-population differences in MTs gene expressions are more visible in the case of liver tissue, where the expression is up to eight times higher (MT II) in polluted sites compared with the unpolluted reference—the Mikołajki site. In contrast, the analysis of MT I expression in the animal testes gave a surprising result of downregulation in animals from all the sites when compared to the Mikołajki site. Contrary to MT I expression, analysis of the testes of animals inhabiting polluted sites and also the Niepołomice site showed a clear pattern of upregulation of the MT II gene (up to 4 times higher than those from the Mikołajki reference site). Taking into account the results of MTs in the liver and MT II in the testes, the results for animals from the Niepołomice site were close to the results for animals from sites initially classified as polluted. These expression data also reflect the results concerning zinc and cadmium levels in these studied tissues.

As mentioned earlier, the statistical results for kidney tissues show a similar level of expression for both the studied MT genes (lower than for Mikołajki) among the studied populations. However, there are some signs of slight upregulation in the tissue of bank voles from polluted sites. In the study by Dondero et al. (2005), the authors assessed gene expression of MT10 and MT20, representing the metallothionein gene family. They found that heavy metals influenced the expression of the MT10 and MT20 genes and the level of expression depended on the metal and gene. In case of cadmium and MT20 expression reached up to 2200-fold induction. However, in most cases MT expression varied between 2- and 30-fold. These results suggest that, in our case, the impact of metal exposure was rather moderate.

Our results concerning MT regulations in the animals from the unpolluted and polluted sites were confirmed by the results of the regression analysis. The regression analysis, which takes into account individual tissue metal concentration and MTs genes expressions, indicated that the metals accumulated in some tissues positively influenced MT I and MT II genes expression (Table 4; Fig. 2). Thus, MT I and II expressions were positively influenced by accumulated zinc and copper in the liver and by lead and cadmium in the kidney. Additionally, MT II expression in the liver was upregulated by cadmium.

Research similar to ours was performed by Fritsch et al. (2010) on seven sympatric small mammal species collected near former smelters along a pollution gradient, in an area considered as highly polluted with Cd, Pb, and Zn. The authors found increasing-with-age Cd concentrations in the liver and kidney in the case of all the studied species and some patterns of low tissue metal concentrations and low metallothioneins levels. A slight increase of MT with Cd accumulation was noted by Fritsch et al. (2010) for bank vole species. The literature provides more examples of MT induction (e.g., the findings of Chater et al. 2008) than inhibition after metal exposure. Chater et al. (2008) studied the effects of sub-acute cadmium treatment (3 mg of cadmium chloride per kg of bw) on pregnant female rats and noted MT increase (measured with Cd method) in the liver tissues. In such a treatment type, MTs induction is supposed to be more evident than after exposure to mostly low environmental contamination, as we hypothesised in our study.

Metallothionein I and II expression in the kidney of Sprague–Dawley rats after intoxication with lead acetate (300 mg/L) and/or cadmium dichloride (50 mg/L), separately and in combination, was studied by Wang and Fowler (2008). As they revealed, there were no effects of lead on both MTs. Animals from the Cd and Cd–Pb treated groups had significantly higher expression in the kidney than from the control and, additionally, animals from the Cd–Pb group had significantly higher MTs expressions than the Cd and Pb separately treated groups, which suggests a synergistic effect of these metals. Despite the fact that our studied populations were exposed to both cadmium and lead, we may only conclude that the main MTs inducer was cadmium because the tissue lead concentration was very low.

According to some literature data rodent testes are more sensitive to cadmium toxicity than the liver tissue (Mckenna et al. 1996; Ren 2003). Available figures are inconclusive about the levels of metallothioneins gene expressions in the testes samples under environmental conditions. In some studies, the authors noted no induction of MT genes expression after Cd exposure (Shiraishi et al. 1995), whereas others confirmed upregulation in rodents intoxicated with Cd and Cd/Zn mixture (Messaoudi et al. 2010). In the studied bank vole testes, the MTs expressions were not high. What more, MT I expression in the testes of animals from the reference site (Mikołajki) was the highest. Downregulation was distinguished for the remaining sites—up to five times lower than at the Mikołajki site. Bonda et al. (2004) showed that testicular MT levels after cadmium intake are lower among adult individuals when compared to young animals. Similar to our research, cadmium contamination seemed to decrease the levels of metallothioneins gene expression. However, in our study, regression analysis did not demonstrate any relationship between metal concentration in the testes and gene expression of MT I, suggesting that this low expression was not due to the studied pollutants but rather that the basal expression was low in all populations due to other potential factors.

## Conclusions

The studied bank vole populations, classified as polluted, were located in areas that have been inundated with metal pollutants for several decades. However, the present investigation of this small mammal did not reveal tissue cadmium and zinc concentrations as high as expected. As known, both metals are the main and strong inducers of metallothionein response in the rodent species. The low metal concentration in the bank vole tissues could cause low metallothionein expressions, only up to few times higher than the reference. Nevertheless, metal exposure-dependent upregulation of both metallothioneins in the livers of bank voles speaks volumes about active defence against contaminants involving the main detoxification process. Similar to other types of defence, MTs induction and MTs protein production are costly and can burden the animal energetic budget, producing negative physiological disturbances. The positive trends for the relationship between tissue metal concentrations and MTs expressions occurring in this study should be taken as a warning, because the increase of environmental contamination may cause an increase of the animal energy budget and, in consequence, problems at organismal and population levels. This could become even more important when animals face several energetically costly stressors.
